# Mixed Dicarboxylic Acids Derived from Polyethylene as a Feedstock for the Synthesis of Polyesters

**DOI:** 10.1021/acssuschemeng.5c04835

**Published:** 2025-10-13

**Authors:** Tom J. Smak, Hugo Aalders, Rijk van Bruggen, Thijs Out, Bram van Rijn, Carmen Ruijs, Rinke Altink, Ina Vollmer, Bert M. Weckhuysen

**Affiliations:** † Inorganic Chemistry and Catalysis Group, Institute for Sustainable and Circular Chemistry, Faculty of Science, 8125 Utrecht University, Universiteitsweg 99, 3584 CG Utrecht, The Netherlands; ‡ 8119 Hogeschool Utrecht, Heidelberglaan 7, 3584 CS Utrecht, The Netherlands; § TNO Brightsite, Urmonderbaan 22, 6167 RD Geleen, The Netherlands

**Keywords:** polyester, dicarboxylic acid, oxidation, chemical recycling, circular polymers, alternative feedstocks

## Abstract

To move away from the currently linear fossil-based plastic value chain, we aim to produce dicarboxylic acid monomers, such as succinic and adipic acid, by the oxidative conversion of polyethylene (PE) wastes. However, a drawback of this technology is that a mixture of dicarboxylic acids of various chain lengths is produced, in contrast to their fossil-based analogs. Therefore, we aim to explore the potential of applying mixed dicarboxylic acids directly in polyester synthesis. The physical properties of these polymers were compared by synthesizing a range of aliphatic polyesters from dicarboxylic acids with a variation in chain length (i.e., C4–C10) and chain length distributions (i.e., 1, 3, 5, and 7 diacids) with 1,4-butanediol as the comonomer. In addition, a polyester was synthesized from a mix of dicarboxylic acids derived from the oxidative conversion of polyethylene (PE). The polymers were characterized with differential scanning calorimetry (DSC), gel permeation chromatography (GPC), X-ray diffraction (XRD), infrared (IR) spectroscopy, nuclear magnetic resonance (NMR), and thermogravimetric analysis (TGA). Using a mixed dicarboxylic acid feedstock enhances the biodegradability but lowers the melting temperature of the polymers made. This can be compensated by the use of a more rigid diol, such as bis-hydroxyethyl terephthalate (BHET).

## Graphical abstract

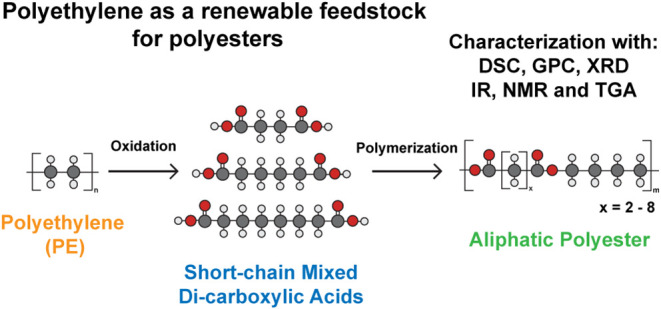

## Introduction

With the incentive to move away from our current linear fossil-based economy, several strategies to produce monomers from more sustainable feedstocks are explored. Dicarboxylic acids monomers, such as succinic and adipic acid, can be produced by the oxidative conversion of polyethylene (PE) waste.^1–3^ Currently, the majority of the adipic acid produced is used for the production of Nylon-6,6.^4^ Succinic acid is used as a monomer in the aliphatic polyester polybutylene succinate (PBS).^5^ Alternatively, a dicarboxylic acid feedstock can be of interest for the synthesis of polyester polyols, which might find wide applications in polyurethanes^6,7^ or as plasticizers.^8^

A drawback of dicarboxylic acids obtained from PE is that a mixture of acids with different chain lengths is obtained, in contrast to their fossil-based analogs. However, with our economy expected to move from a linear one toward a more circular one, it would be of great interest to explore the potential of applying these new feedstocks, from plastic waste or, alternatively, biomass,^9,10^ directly to simplify complex separation procedures. This could also be helpful in designing product purification targets for the oxidative recycling of polymers. At the moment, mixtures of succinic, glutaric, and adipic acid (mainly succinic and glutaric) produced as a side product from adipic acid synthesis have a market and are sold in their methyl ester form as a green solvent.^11^ However, preferably these dicarboxylic acid mixtures can directly be applied in higher value products, such as in polymers.^5^ As the product group of interest we selected aliphatic polyesters, which typically find application in packaging, agricultural films, fibers, elastomers, and coatings.^5,12^ We hypothesized that a mixed feedstock might be good enough for these applications and that because of the mixture the biodegradability could be enhanced.

Although, copolymers synthesized with two different dicarboxylic acids are a well-studied topic (e.g., polybutylene succinate-*co*-adipate),^13,14^ more complex mixtures are rarely studied,^10,15–17^ with only one example studying the effect of the composition of the dicarboxylic acid mixture.^18^ Nelson et al. synthesized a series of aliphatic polyesters using mixtures of ethylene glycol and long–chain dicarboxylic acids with an average chain length varying from C11 to C21. With mixtures of dicarboxylic acids, they observed that an increasing number of different chain lengths, referred to as chain length distribution, lowers the melting temperature. In addition, effects related to using odd or evenly numbered chain lengths disappear. With a mixture off five dicarboxylic acids centered around C14, they obtained mechanical properties in between the properties of high-density polyethylene (HDPE) and low-density polyethylene (LDPE). When they increased the number of dicarboxylic acids to 17 different carboxylic acids centered around C12, they observed that the material becomes softer and more brittle.^18^

The results of Nelson et al. show that it is possible to produce strong polyesters with PE-like mechanical properties when using long-chain dicarboxylic acids in the mixed form.^18^ In addition, Klingler et al. has demonstrated that diacids obtained from HDPE can be repolymerized.^19^ Despite these interesting findings, there is still a mismatch between the most advanced oxidative recycling methods and the polymers produced. The higher yielding PE oxidation methods use HNO_3_ ^1,15,20^ as an oxidant or are variations of the conditions used for the Amoco Mid Century Technology, which employs Co/Mn/Br as a catalyst and is performed in acetic acid with O_2_.^2,21^ Both methods result in mainly short-chain dicarboxylic acids (C4–C7) up to ∼40 mol % yield. Longer-chain dicarboxylic acids are also accessible, albeit in relatively small amounts.

In this work, we aim to demonstrate the potential and limitation of a mixed short dicarboxylic acid feedstock (Figure 1). This was achieved by synthesizing a series of polyesters using 1,4-butanediol and dicarboxylic acids (C4–C10) with varying chain length distributions, which were characterized with differential scanning calorimetry (DSC), gel permeation chromatography (GPC), X-ray diffraction (XRD), infrared (IR) spectroscopy, and thermogravimetric analysis (TGA). In addition, an experiment was performed with dicarboxylic acids derived from the oxidative conversion of HDPE. The biodegradability of polymers synthesized with varying number of different dicarboxylic acids was tested in line with ASTM method D5988–18 for determining aerobic biodegradation of plastic materials in the soil. At last, some experiments were performed with bis-hydroxyethyl terephthalate (BHET) as a diol, which might be a way to overcome the limitations of low temperature melting transitions of polyesters produced from short-chain dicarboxylic acids.

**Figure 1:**
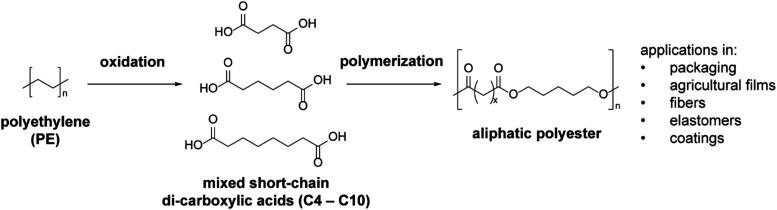
Polyethylene (PE) can be oxidatively converted to mixtures of short-chain dicarboxylic acids, which can serve as the feedstock for the synthesis of polyester materials.

## Results and Discussion

To study the effect of chain length and the number of different dicarboxylic acids on the polymer properties, a series of polymers was prepared through a two-step polycondensation with 1,4-butanediol (Figure 2a).^5^ 1,4-butanediol was selected because of its widespread use in important aliphatic (co)­polyesters (e.g., PBS, polybutylene succinate adipate (PBSA) and polybutylene adipate terephthalate (PBAT)).^12^ First, an oligomerization step was performed at 150 °C under an N_2_ atmosphere. In the second step, the temperature was increased to 250 °C under vacuum to obtain the desired polymer. The produced polyesters were named PE-4,*X* ± *Y*, where *X* is the chain length of the dicarboxylic acid center and *Y* is the range of the dicarboxylic acid distribution. For the experiments with mixed diacids, the following chain length distributions were used: 1:2:1 (*Y* = 1), 1:2:3:2:1 (*Y* = 2), and 1:2:3:4:3:2:1 (*Y* = 3). A table with the dicarboxylic acid compositions used for each polymer can be found in the Supporting Information (SI, Section 2). The degree of polymerization was determined by GPC. Number-average molecular weight (*M_n_*) values ranging from 5400 to 22,300 g/mol were observed, and the exact numbers can be found in the SI (Section 1). The observed variations are probably the result of a difference in monomer purity, which was typically a bit lower for the uneven dicarboxylic acids. Nevertheless, all molecular weights are in a range where the expected influence on the properties studied below is small.

**Figure 2:**
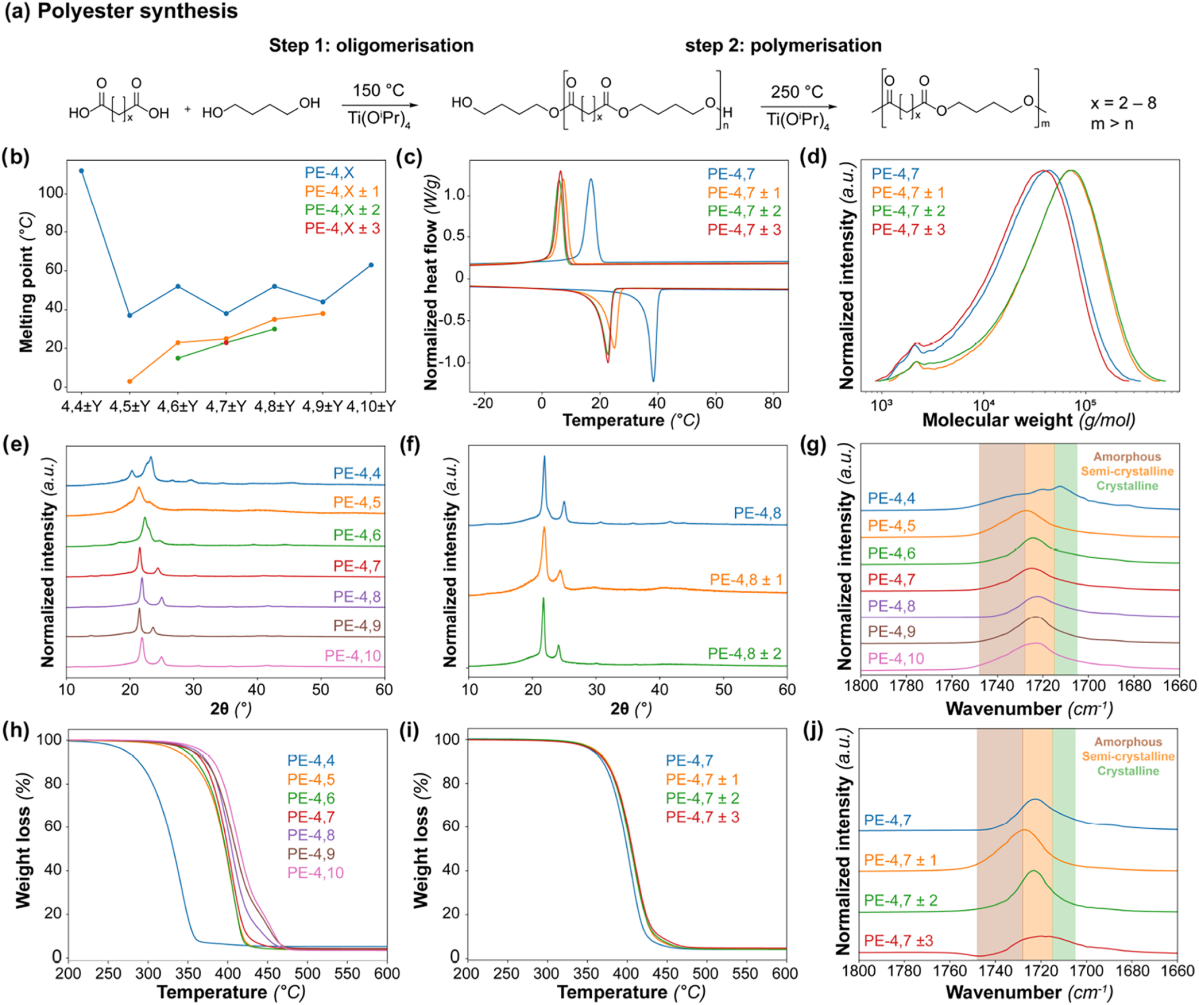
(a) The polymerization experiments of the desired dicarboxylic acid mixture with 1,4-butanediol were performed in two steps. (1) First, an oligomerization step of the diacid and excess diol (1.1 equiv) was performed at 150 °C under an N_2_ atmospheric pressure. (2) In the second step, the temperature was increased to 250 °C under vacuum to obtain the desired polymer. (b) The melting transitions of the synthesized polyesters obtained with differential scanning calorimetry (DSC), both as a function of dicarboxylic acid chain length and chain length distribution. (c) DSC curves (exo up) of PE-4,7 as a function of chain length distribution. (d) Gel permeation chromatography (GPC) profiles of the PE-4,7 as a function of dicarboxylic acid chain length. (e) X-ray diffraction (XRD) pattern of PE-4,*X* as a function of dicarboxylic acid chain length. (f) XRD pattern of PE-4,8 as a function of chain length distribution. (g) Infrared (IR) spectra of polyester with composition PE-4,*X* as a function of the diacid chain length. (h) Thermogravimetric analysis (TGA) data of polyesters with the composition PE-4,*X* as a function of diacid chain length. (i) TGA data of polyesters with the composition PE-4,7 ± *Y* as a function of chain length distribution. (j) IR spectra of polyesters with the composition PE-4,7 ± *Y* as a function of chain length distribution.

The solid-state structure and the related melting properties were studied with DSC, XRD, and IR spectroscopy. The melting temperatures were determined with DSC and, for the series PE-4,*X* ± 0, values ranging from 37 to 112 °C were observed (Figure 2b). For all polymers, the melting transitions match with the literature values,^22–25^ except for PE-4,7 as no literature value could be found. The temperature of the melting transition of PBS (PE-4,4) was significantly higher compared to the other polymers, namely, 112 °C. For the remaining series, an increase in melting temperature was observed toward longer dicarboxylic acid chain lengths, with the even dicarboxylic acids having a higher melting temperature than the uneven dicarboxylic acids, which is referred to as odd/even effect.

The melting temperatures decrease upon an increasing number of dicarboxylic acids, and the value plateaus around a value that seems mainly determined by the average chain length. In addition, the odd/even effects disappear with increasing number of different dicarboxylic acids. This is further illustrated by the DSC curves of PE-4,7 ± *Y* (Figure 2c), that show a decrease in melting temperature for an increase in *Y*. Similar trends were observed for other dicarboxylic acid chain lengths, and plots for PE-4,6 ± *Y* and PE-4,8 ± *Y* can be found in Figure S8. The decrease in melting temperature and the disappearance of the odd/even effect at increasing number of dicarboxylic acids were observed by Nelson et al. for dicarboxylic acid mixtures centered around C11 to C22.^18^ Furthermore, it is interesting to note that the enthalpy of melting did not change upon an increase of the number of different dicarboxylic acids and nearly identical values were found for PE-4,7 ± *Y* (51–56 J/g). This indicates a similar crystallinity for polyesters produced from a mixed feedstock compared to that from a single dicarboxylic acid feedstock. Similar trends in melting temperature and enthalpy of melting were observed for the other polymers, except for PE-4,5 ± 1, which is a nearly amorphous polymer (exact values in the SI, Section 1).

The observations made with DSC were confirmed with XRD (Figure 2e,f). Furthermore, XRD revealed that polymers prepared from dicarboxylic acids centered around C7 or higher adopt an orthorhombic structure resembling characteristics of HDPE, revealing the alignment of the methylene units in the backbone.^18^ In addition, PE-4,4 and PE-4,6 were both present in the α-form.^26,27^ Due to the relatively low melting temperature of most polyesters, it was not possible to record an XRD pattern for all polyesters synthesized from mixed dicarboxylic acids (PE-4,5 ± *Y*, PE-4,6 ± *Y*, and PE-4,7 ± *Y*). The XRD patterns of PE-4,8 ± *Y* revealed only a minor decrease in crystallinity upon the increasing number of dicarboxylic acids, but the crystal structure is retained.

The ordering within the polyesters as a function of dicarboxylic acid chain length and distribution was studied in more detail with IR spectroscopy (Figure 2g,j). The peak location of the vibration corresponding to the carbonyl is a measure of the degree of dipole–dipole interactions between the different carbonyl groups (δ^–^ → δ^+^), with a lower energy corresponding to a stronger interaction. Therefore, the location provides information about ordering of the material. In the carbonyl region of PBS (PE-4,4), we observe three different peak maxima located at ∼1736, ∼1720, and ∼1714 cm^–1^, which can be assigned to a free amorphous fraction, a rigid amorphous fraction, and a crystalline fraction, respectively.^28^ For all other polymers, only one peak maximum in the carbonyl region was obtained, with the peak maximum shifting from ∼1730 cm^–1^ for PE-4,5 to ∼1722 cm^–1^ for PE-4,10. This shows that the solid-state structure changes from nearly amorphous to a semiorganized structure upon increasing dicarboxylic acid chain length, which correlates with the XRD data. In addition, odd polymers have a peak at a slightly higher wavenumber than even polymers, indicating less dipole–dipole interactions (e.g., less ordering). The IR spectra of PE-4,7 ± *Y* (Figure 2j) show a shifting location of the carbonyl peak maximum (∼1723, ∼1727, ∼1724, and ∼1720 cm^–1^) with increasing number of different dicarboxylic acids. This nonlinear shift in the wavenumber suggests that the alignment in ester groups first decreases but that a further increase in chain length distribution allows for better alignment. Nevertheless, the differences are small, and all polymers seem to have a semicrystalline structure. Overall, we can conclude that the use of a mixed dicarboxylic acid feedstock lowers the melting temperature. In addition, the effect of using multiple dicarboxylic acids is less pronounced when the dicarboxylic acid chain length is longer. This result can be attributed to a relatively smaller disturbance of the crystal structure with an increasing number of methylene units.

The thermal stability as a function of dicarboxylic acid chain length was studied with TGA (Figure 2h). The temperature at which 5 wt % of the polymer has decomposed (*T*_5%_) matches the literature values for the known polymers (PE-4,4, PE-4,6, PE-4,9, and PE-4,10), and detailed tabulated data can be found in the SI (Section 1 and Table S1).^23,25^ The decomposition temperature increases with the number of methylene units in the dicarboxylic acid chain length, suggesting that the thermal stability mainly depends on the density of ester bonds, which are more easily broken. The TGA curves for PE-4,7 ± *Y* as a function of chain length distribution (Figure 2i) show that the decomposition temperature does not change upon an increase in the number of different dicarboxylic acids. The same invariance was observed for the other dicarboxylic acid chain lengths (Figure S8). This is in line with the observation in Figure 2h, where the decomposition temperature seems to be mainly dependent on the density of ester groups.

In our laboratory, some attempts were made to study the mechanical properties of our materials. Unfortunately, we did not succeed in making the required test specimen, both for the reference materials with one dicarboxylic acid (e.g., PE-4,4, PE-4,6, and PE-4,10) and the samples with multiple dicarboxylic acids (e.g., PE-4,6 ± 1, PE-4,9 ± 1), because all samples were too brittle. It was either due to the properties of our materials or due to the unavailability of equipment to make a thin homogeneous film under an inert atmosphere.

Aliphatic polyesters are well-known for their biodegradability.^29^ Therefore, the effect of the number of different dicarboxylic acids was studied on a few selected polymers. The biodegradation experiments were performed using the soil burial method ASTM D5988–18, and the mineralization rate was determined by titration.^30^ The experiments were performed on PE-4,6 ± *Y* with varying chain length distributions (*Y* = 0, 1, and 2) (Figure 3). The three polymers had a comparable molecular weight (SI, Section 4) and crystallinity (DSC), thereby excluding effects that are not related to a mixed feedstock. For the polymers prepared from multiple different dicarboxylic acids, an increase in mineralization rate was observed compared to the nonmixed polymer. After 90 days, approximately 16 wt % of PE-4,6 was mineralized, compared to 49 and 67 wt % for PE-4,6 ± 1 and PE-4,6 ± 2, respectively (Figure 3a). The enhancement in biodegradation is further illustrated in Figure 3b,c with photographs of the polyester samples taken before and after 90 days in the soil. The polyester PE-4,6 was still largely intact and had become a bit yellowish compared to the virgin sample, while PE-4,6 ± 1 was visually affected to a large extent and had become yellow-brown-greenish and very brittle. In addition, no leftover material of PE-4,6 ± 2 was observed after 90 days in the soil. For the latter sample, its melting transition around room temperature can possibly explain why the small percentage of nonmineralized materials was not recovered after the experiment. For the control experiment with PE-4,6 ± 2 without soil, no decomposition was observed, and the sample looked visually similar after 3 months. The results shown in Figure 3 clearly demonstrate that applying a mixed dicarboxylic acid feedstock enhances the biodegradability.

**Figure 3:**
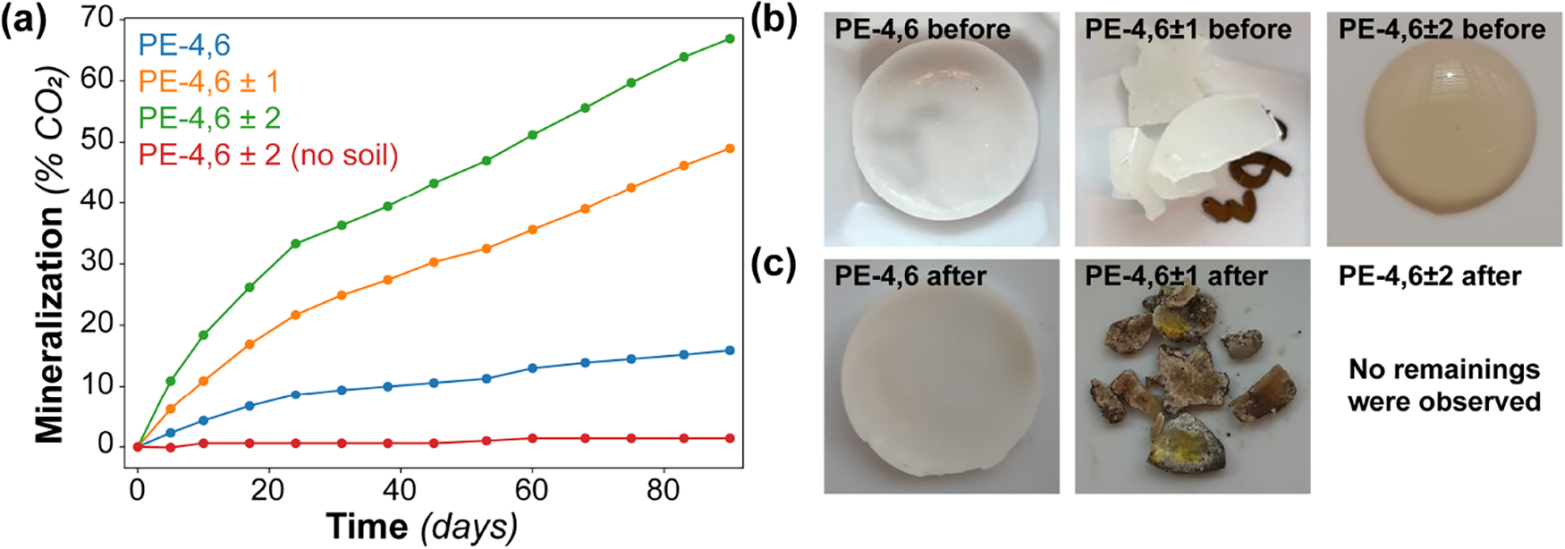
(a) Biodegradation of the polyester PE,4,6 ± *Y* as a function of time in a soil burial experiment. (b) The polyester samples PE-4,6, PE-4,6 ± 1, and PE-4,6 ± 2 before the soil burial experiment. (c) The polyester samples PE-4,6 and PE-4,6 ± 1 after 90 days of being buried in the soil. No leftover material of PE-4,6 ± 2 was observed after 90 days, while PE-4,6 ± 2 without soil looked identical to the initial state after 90 days.

Next, a polymer was produced with dicarboxylic acids obtained from HDPE. First, HDPE was oxidized at 130 °C for 16 h at a pressure of 30 bar synthetic air.^3^ Due to the small reaction scale and complexity of the product mixture, instead of classical purification methods, a consecutive oxidation step using 65% HNO_3_ was performed. In this step, partially oxidized functionalities and undesired end groups (e.g., methyl ketone, γ-lactone)^3^ were converted to carboxylic acids. Reaction and purification were performed twice, where one batch was used for polymerization with 1,4-butanediol and the other for product analysis with a gas chromatograph (GC) equipped with a flame ionization detector (FID). An isolated dicarboxylic acid yield of 12 mol % was obtained. The yield is defined as the percentage of carbon that ends up in the dicarboxylic acid product. Higher dicarboxylic acid yields up to 30–40 mol % can be accessed,^1,21^ but suitable purification methods still need to be developed. In Section 11 of the SI, preliminary data on how the dicarboxylic acid chain length can be tuned toward longer dicarboxylic acids with an Mn catalyst or toward shorter chain lengths with the addition of NO to the reaction atmosphere. Crucial parameters to optimize the yield are also discussed. These results will be expanded in future publications, while the focus in the current study was the polymerization step.

GC-FID analysis of the oxidized HDPE sample showed the formation of a dicarboxylic acid series with chain lengths ranging from C4 to C20 (Figure 4b). These dicarboxylic acids were polymerized, and the end group analysis with nuclear magnetic resonance (NMR) revealed an *M_n_* of 5580 g/mol (Figure 4c). This is lower compared to the same experiment performed with standard dicarboxylic acid mixtures and this is likely the result of trace amount of undesired end groups (e.g., alkyl, methyl ketone, and γ-lactone).^3^ In addition, an average dicarboxylic acid chain length of 7.45 was calculated based on NMR, which is in agreement with the GC-FID analysis. Furthermore, IR spectroscopy confirmed successful polymerization (SI, Section 8), and the C=O stretch vibration was located at ∼1728 cm^–1^, indicating a nearly amorphous polymer.^28^ This was confirmed by the DSC analysis, showing a melting transition at −9 °C, and the enthalpy of melting was significantly lower compared to the polymers studied before (SI, Section 8). The differences between the simulated mixtures and the diacids derived from HDPE highlight the importance to look at real samples. The larger variation of the number of different dicarboxylic acids in the real sample, combined with small amounts of impurities obtained in HDPE oxidation are likely responsible for the differences in melting temperature and crystallinity. It is important that suitable purification methods are being developed, and possibly fractionation of the dicarboxylic acid mixture is required to improve the polymer properties.

**Figure 4:**
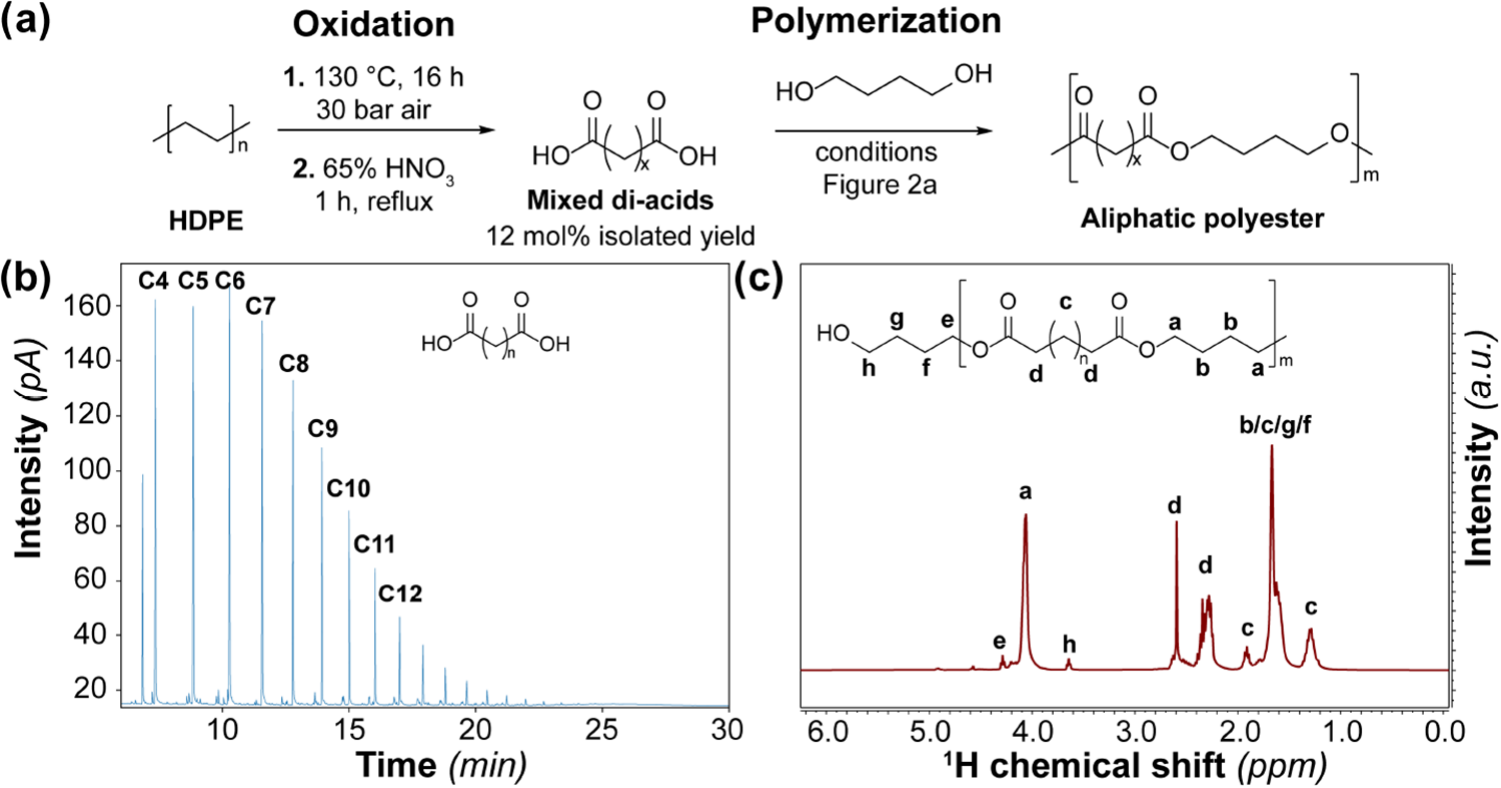
(a) Overview scheme showing the two-step high-density polyethylene (HDPE) oxidation and the polymerization step of the HDPE-derived dicarboxylic acids. (b) Gas chromatography–flame ionization detection (GC-FID) plot of a dicarboxylic acid mixture obtained from HDPE through a two-step oxidation procedure. (c) ^1^H nuclear magnetic resonance (NMR) spectrum of the polyester synthesized from the dicarboxylic acid mixture shown in panel (a). A larger figure with a more extended assignment can be found in the Supporting Information (SI, Figure S14).

Since, the melting temperatures of the polyesters with a large distribution in dicarboxylic acids are too low to be directly used in polymer applications, we used the commercially produced polymer poly­(butylene-adipate-*co*-terephthalate) (PBAT),^31^ as inspiration to overcome this limitation. In PBAT, part of α,ω-diacid is replaced by terephthalic acid, leading to a combination of the good mechanical properties of an aromatic polyester with an excellent biodegradability of an aliphatic polyester.^31^

In the experiments, 1,4-butanediol was replaced with bis-hydroxyethyl terephthalate (BHET), which can be obtained from the polyethylene terephthalate (PET) waste through glycolysis.^32^ According to the standard synthesis procedure, two polymers with the compositions PE-BHET,6 ± 2 and PE-BHET,8 ± 2 were synthesized (Figure 5). The DSC analysis showed that both polymers were nearly completely amorphous, but a relative broad phase transition assigned to the melting transition was observed at 134 °C for PE-BHET,6 ± 2 and 123 °C for PE-BHET,8 ± 2 (Figure 5b). As these phase transitions were not very pronounced, these values were also confirmed with a melting point apparatus, and the values of 130 and 106 °C were found. In addition, a glass transition temperature (*T*_g_) of −1 °C for PE-BHET,6 ± 2 and −7 °C for PE-BHET,8 ± 2 was observed. XRD analysis confirmed that the polymer was mostly amorphous (Figure 5c), and it was observed that the XRD pattern was similar to that of PBAT.^33^ In addition, TGA showed a *T*_5%_ of 354 °C for PE-BHET,6 ± 2 and 386 °C for PE-BHET,8 ± 2 (SI, Figure S11). The XRD and DSC data of our polymers were comparable to the aliphatic-aromatic copolyester reported by Nelson et al., who studied the effect of the aromatic content on longer dicarboxylic acids.^17^ Based on these observations, it can be concluded that the use of a short-chain mixed dicarboxylic acid feedstock has a smaller influence in aliphatic-aromatic copolyesters than in purely aliphatic polyesters, possibly due to the already nearly amorphous nature of aromatic copolyesters. In addition, it can be concluded that with mixed dicarboxylic acids, materials with similar characteristics as the ones of PBAT can be obtained, when an aromatic diol is used.

**Figure 5:**
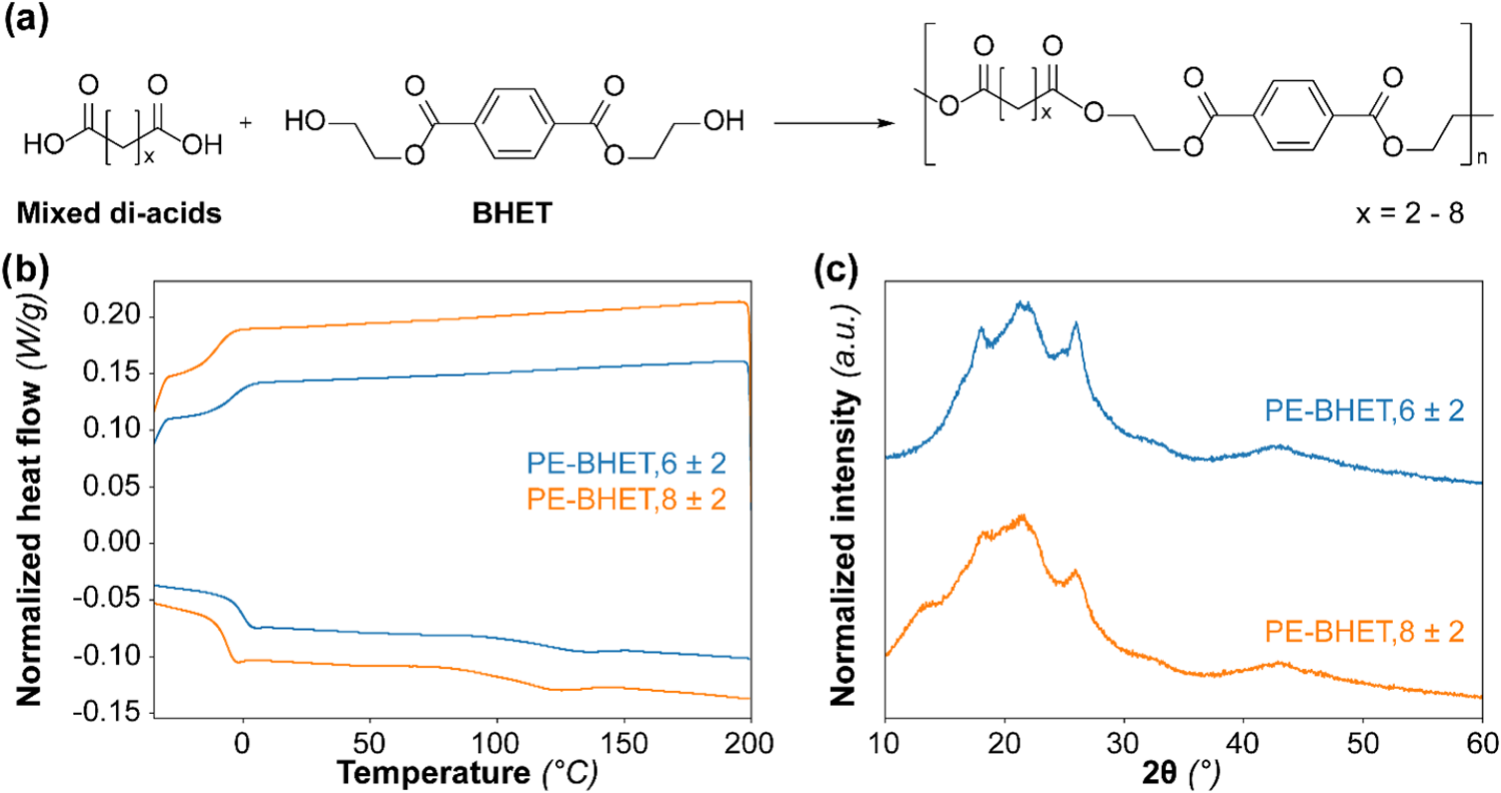
(a) Mixed dicarboxylic acids were successfully polymerized with bis-hydroxyethyl terephthalate (BHET) to obtain an aliphatic-aromatic-*co*-polyester. (b) Differential scanning calorimetry (DSC) curves of the polyesters produced with BHET (exo up). (c) X-ray diffraction (XRD) patterns of the polyesters produced with BHET.

## Conclusions

Polyesters can be synthesized using 1,4-butanediol together with a mixed dicarboxylic acid feedstock obtained from the oxidation of (PE), as well as from various dicarboxylic model compound mixtures. Differential scanning calorimetry (DSC) analysis showed that an increase in the number of different dicarboxylic acids results in a decrease in the melting temperature of the corresponding polyesters. For the polymers with a dicarboxylic acid distribution centered around C7, an orthorhombic structure resembling the characteristics of high-density polyethylene (HDPE) was observed, revealing the alignment of the methylene units in the backbone. Upon an increase of the number of different dicarboxylic acids, no significant differences in crystallinity were observed with X-ray diffraction (XRD) and the enthalpy of melting obtained with DSC. Furthermore, it was observed with thermogravimetric analysis (TGA) that the *T*_5%_ value mainly depends on the dicarboxylic acid chain length, with the longer dicarboxylic acids decomposing at a higher temperature. An increase in the number of dicarboxylic acids did not affect the *T*_5%_ values. Furthermore, it was observed that the biodegradability of the polyesters made can be significantly enhanced through the use of a mixed dicarboxylic acid feedstock. In addition, polyethylene (PE) was successfully converted to a dicarboxylic acid mixture, which was polymerized and further characterized.

A limitation of the polymers produced from 1,4-butanediol and mixed dicarboxylic acids is that the melting temperatures are too low for a viable application. Introducing an aromatic ring is introduced by replacing 1,4-butanediol with BHET, this limitation can be overcome, and the melting temperatures of the corresponding polymers can be significantly increased. This yields a polymer with similar characteristics as PBAT. However, further research is required on how the mechanical properties change upon applying a mixed dicarboxylic acid feedstock in combination with an aromatic diol; this should also provide more information on which chain length and distribution oxidation of PE one should aim for. Currently, the main challenge for PE oxidation seems to be the quality of the dicarboxylic acid feedstock, and research should focus on narrowing the diacid distribution and decreasing the amount of impurities. A higher feedstock quality can potentially be achieved by finding new and better catalyst formulations. When this is not possible, it is recommended to develop a technology similar to adipic acid purification, where the majority of the valuable compounds is extracted and the remaining is sold as a mixture.

## Experimental Section

### Materials and Reagents

The following reagents were purchased and used as received, unless noted otherwise: succinic acid (Sigma-Aldrich, >99%), glutaric acid (Sigma-Aldrich, 99%), adipic acid (ABCR, 99%), pimelic acid (Thermo Scientific, 98%), suberic acid (Sigma-Aldrich, 98%), azaleic acid (Acros Organics, 99%), sebacic acid (Sigma-Aldrich, 99%), 1,4-butanediol (Sigma-Aldrich, 99%), Ti­(OiPr)_4_ (Acros Organics/Sigma-Aldrich, 98/97%), chloroform (Biosolve, HPLC grade), bis-hydroxyethyl terephthalate (Sigma-Aldrich, >94.5%), HDPE (Sabic, *M*_w_ = 137,500 g/mol, *M_n_* = 16,010 g/mol), methanol (Fisher Scientific, HPLC grade), HNO_3_ (VWR Chemicals, 65%), acetyl chloride (Sigma-Aldrich, >99%), 4-heptanone (Across Organics, >98%) HCl (Supelco, 37%), KOH (Sigma-Aldrich, 85%), phenolphthalein (Fisher Scientific), and CDCl_3_ (Cambridge Isotope Laboratories, D 99.8%).

### Analytical Methods

Differential scanning calorimetry (DSC) measurements were performed on a TA DSC 2500 instrument using the following temperature program: heating to 150 °C with 5 °C/min and then keeping the sample at 150 °C for 5 min. Subsequently, the sample was cooled to −40 °C with a ramp of 5 °C/min and kept at −40 °C for 5 min. The second heating cycle was performed with a temperature ramp of 5 °C/min to 150 °C. For analysis, the second heating cycle and the first cooling cycle were used.

X-ray diffraction (XRD) measurements were performed on a Bruker D2 phaser second generation powder X-ray diffractometer. The data were recorded in the Bragg mode using a Cu Kα (λ = 1.54 Å) radiation source. Prior to XRD analysis, the solid polymer was cut and broken into as small as possible pieces. Subsequently, the samples were pressed into a powder XRD sample holder. Fourier transform (FT) infrared (IR) spectra were recorded on a PerkinElmer FT-IR Frontier spectrometer with a PerkinElmer Universal attenuated total reflectance (ATR) sampling accessory and mercury cadmium and telluride (MCT) detector. The spectra (8 scans) were recorded in the ATR mode in the range 4000–600 cm^–1^ with a resolution of 1 cm^–1^. The samples were measured without any further sample preparation, and the solids were pressed against the ATR crystal using the clamping arm.

Thermogravimetric analysis (TGA) was performed on a PerkinElmer TGA 8000 instrument. The TGA data were recorded under N_2_ using a temperature ramp of 10 °C/min from 50 to 600 °C.

Nuclear magnetic resonance (NMR) experiments were measured on an Agilent MRF 400 instrument equipped with a OneNMR probe and an Optima Tune system. The NMR spectra were recorded in CDCl_3_ and the resonances were referenced to the residual solvent peak (^1^H: δ 7.26 ppm for CDCl_3_).

Gas chromatography (GC) was performed on a Thermo Scientific Trace GC 1300 instrument equipped with a total ion chromatogram (TIC) and a flame ionization (FID) detector. Samples were injected at an injector temperature of 250 °C. The products were separated using a GC 30 m, 0.25 mm ID, 0.25 m column. The column oven temperature was kept at 40 °C for 5 min, increased at 12 °C/min to 320 °C, and held for 20 min.

Gel permeation chromatography (GPC) experiments were performed in chloroform at 35 °C on a PSS SECcurity2 instrument, equipped with PSS SDV linear M columns (2 × 30 cm^2^, additional guard column) and a refractive index detector (PSS SECcurity2 RI). The standard flow rate used was 1 mL/min. The molecular weights were determined versus narrow polystyrene samples (software: PSS WinGPC, version 8.32).

Melting transition measurements were performed on a Büchi Model M-560 melting point apparatus.

### General Synthesis Procedure for Aliphatic Polyesters

The synthesis procedure was adapted from Platnieks et al.^5^ In the following order, dicarboxylic acid (1 equiv), 1,4-butanediol (1.1 equiv), and Ti­(O^i^Pr)_4_ (0.02 equiv) were added into a round-bottom flask. Subsequently, the round-bottom flask was evacuated three times and refilled with N_2_ to remove all O_2_ present. Then, the reaction mixture was heated under N_2_ to 150 °C and held for 45 min. In the next hour, every 15 min the system was evacuated to 5 mbar for 5–10 s. At last, the round-bottom flask was heated up to 250 °C and left there under vacuum for 3 h. Then, the polymer was cooled to room temperature under an N_2_ atmosphere. Typical experiments were performed to obtain about 2 g of polymer, and the materials were directly used as synthesized. The aliphatic-aromatic copolyesters were prepared using the same experimental procedure but replacing 1,4-butanediol by bis-hydroxyethyl terephthalate (BHET).

### Biodegradability Experiments

ASTM method D5988–18 for determining the aerobic biodegradation of plastic materials in the soil was used as a guideline for the biodegradability experiments.^30^ The biodegradability experiments were performed on PE-4,6, PE-4,6 ± 1, and PE-4,6 ± 2, which were synthesized according to the standard protocol mentioned above without a catalyst and molten into a disk shape. In addition, a soil blank and an experiment using PE-4,6 ± 2 without soil were performed.

Soil for the experiments was collected at three distinct locations with the following coordinates: 52°06′51.9″N 5°05′53.4″E, 52°06′57.7″N 5°05′43.4″E, and 52°06′26.2″N 5°06′55.6″E. Subsequently, the soil was sieved, such that only particles smaller than 2 mm were obtained. Equal amounts by weight from each location were mixed, and the pH of the soil was neutral. The moisture content was 39% (determined by weighing after drying). Subsequently, 500 g of soil and the 0.4–0.8 g polyester were placed in a desiccator (SI, Figure S1). On the top of the soil, a beaker with 20 mL of 0.5 M KOH was placed. Once in every week, the beaker with KOH was titrated with 0.25 mL of HCl and replaced for a new beaker. The titrations were performed using the pH indicator phenolphthalein, which loses the pink color below a pH of 8.2 In the SI, Section 3, all formulas to calculate the amount of released carbon and the tables with the measured values are shown.

### Dicarboxylic Acid Production from Polyethylene

The oxidative conversion of polyethylene (PE) was performed in two steps. The first step was performed batchwise in a Parr autoclave of 50 mL similar to the experiments performed in the previous work from our group.^3^ In the experiment, a borosilicate glass liner was filled with ∼200 mg of HDPE, which was placed in the reactor. Before the experiment, the reactor was purged with N_2_. Then, the vessel was pressurized with 30 bar of synthetic air (O_2_/N_2_:20/80). The pressures are reported at room temperature. The HDPE layer in the bottom of the reactor was too thin to apply stirring. Subsequently, the autoclave was heated up to 130 °C for 16 h. The reactor needed 1 h to reach the final temperature and when the final temperature was reached the reaction time was set to zero. After the desired temperature program was completed, the reactor was allowed to cool down to room temperature.

The second oxidation step was performed for purification with HNO_3_. To the complete reaction mixture obtained from the reactor, 2 mL of 65% HNO_3_ were added into the glass liner, and the mixture was heated for 1 h to 100 °C, followed by an evaporation of the HNO_3_ until a white solid was obtained. This white solid was either used directly for polymerization according to the standard procedure or for analysis with GC-FID.

Prior to GC analysis, the product mixture was esterified with a mixture of 6 mL of acetyl chloride/methanol (1:20; v/v) at 50 °C for 1 h, yielding a clear solution. Subsequently, a drop of 4-heptanone was added as an internal standard. As a precaution to prevent the GC from clogging, the sample was filtered with a 0.45 μm filter. Weighing the filter reveals that an insignificant amount is filtered off. Response factors for GC-FID were calculated using effective carbon number (ECN) theory.^34,35^ The dicarboxylic acid yield was 41 mg (12 mol %). The yield is defined as the percentage of carbon from PE that ends up in the dicarboxylic acid product (eq 1).

1 $$yield\;\left(mol\;\%\right)=\frac{C_{di‐acids}}{C_{PE}}\times 100\%$$

## Supplementary Material



## Data Availability

All data and python scripts utilized in the manuscript have been uploaded to the YODA repository and are available under 10.24416/UU01-CX4YPC.

## Notes

Details on the setup and example calculations for the biodegradability experiments, characterization of the materials used for biodegradation, additional characterization on the polyesters derived from HDPE and BHET and an outlook on PE oxidation. In addition, it contains an overview of the available raw data (PDF)

## Abbreviations

PE: polyethylene
HDPE: high-density polyethylene
DSC: differential scanning calorimetry
GPC: gel permeation chromatography
XRD: X-ray diffraction
IR: infrared
NMR: nuclear magnetic resonance
TGA: thermogravimetric analysis
BHET: bis-hydroxyethyl terephthalate
PBS: polybutylene succinate
ATR: attenuated total reflectance
MCT: mercury cadmium and telluride
GC: gas chromatography
TIC: total ion chromatogram
FID: flame ionization detector
PBAT: polybutylene-adipate terephthalate
PET: polyethylene terephthalate
