# Supplementary material for: Mixed Dicarboxylic Acids Derived from Polyethylene as a Feedstock for the Synthesis of Polyesters
Source: ACS Sustain Chem Eng. 2025 Oct 13;13(42):17806–14. doi: 10.1021/acssuschemeng.5c04835 (PMC12570260; doi:10.1021/acssuschemeng.5c04835)
Supplement: Supplementary file 1 [file sc5c04835_si_001.pdf]

Supporting Information

Mixed Di-Carboxylic Acids Derived from Polyethylene as a Feedstock for the Synthesis of Polyesters

*Tom J. Smak,[a] Hugo Aalders,[a] Rijk van Bruggen,[b] Thijs Out,[b] Bram van Rijn,[b] Carmen Ruijs,[b] Rinke Altink,[c] Ina Vollmer[a,] *, and Bert M. Weckhuysen[a,] **

a) Inorganic Chemistry and Catalysis group, Institute for Sustainable and Circular Chemistry, Faculty of Science, Utrecht University, Universiteitsweg 99, 3584 CG Utrecht, The Netherlands

b) Hogeschool Utrecht, Heidelberglaan 7, 3584 CS Utrecht, The Netherlands

c) TNO Brightsite, Urmonderbaan 22, 6167 RD Geleen, The Netherlands

Number of pages = 12

Number of figures = 15

Number of tables = 2

Table of Contents

Section	Content	Page
1.	Summary of all data used in the manuscript	3
2.	Di-carboxylic acid compositions used for each polymer in the manuscript	3
3.	Biodegradability setup and calculations	4
4.	Characterization of the materials used for biodegradation	5
5.	Gel permeation chromatography data	7
6.	Differential scanning calorimetry and thermogravimetric analysis plots PE-4,6±Y and PE-4,8±Y	8
7.	Infrared spectra of the polyesters as function of chain length and mixing degree	9
8.	Additional characterization of the polyester derived from HDPE	9
9.	Additional characterization polyesters prepared with BHET	10
10.	Detailed NMR assignment of the polyester derived from polyethylene	11
11.	Polyethylene oxidation: tuning di-carboxylic acid chain length and improving yield	11

1. Summary of all data used in the manuscript

Table S1. Summary of the properties of all polyesters that were discussed in the manuscript. The majority of numbers mentioned below is plotted in Figure 3, 4, and 7.

Entry	Polymer	Tm (˚C)	Tc (˚C)	ΔH (J/g)	$T_{5\%}$ (˚C)	M_n (g/mol)	M_w (g/mol)	D
1.	PE-4,4	112	72	39.3	273	16441	79509	4.84
2.	PE-4,5	37	-1	38.1	337	8568	25171	2.94
3.	PE-4,6	52	31	49.1	348	18909	62015	3.28
4.	PE-4,7	38	17	53.3	356	13829	40646	2.94
5.	PE-4,8	52	36	80.9	361	11459	33351	2.91
6.	PE-4,9	44	28	64.6	356	6392	19655	3.07
7.	PE-4,10	63	44	84.4	369	12000	39348	3.28
8.	PE-4,5±1	3	~-30	9.8	325	15717	70170	4.46
9.	PE-4,6±1	23	2	43.5	347	14542	40426	2.78
10.	PE-4,7±1	25	7	53.2	361	24108	70753	2.93
11.	PE-4,8±1	35	17	58.1	365	18121	53500	2.95
12.	PE-4,9±1	38	34	68.2	364	9623	31140	3.24
13.	PE-4,6±2	15	-8	44.3	339	11238	35668	3.17
14.	PE-4,7±2	23	6	51.2	359	22328	72328	3.23
15.	PE-4,8±2	30	15	63.7	361	10100	29343	2.95
16.	PE-4,7±3	23	6	56.1	358	12328	35501	2.88
17.	PE-BHET,6±2	134	99	0.1	354	5417	12224	2.25
18.	PE-BHET,8±2	123	84	0.1	386	10494	28658	2.73

2. Di-carboxylic acid compositions used for each polymer in the manuscript

Table S2. An overview of the di-carboxylic composition used for each polymer in the manuscript. In all experiment, the diol was 1,4-butanediol, except when indicated otherwise.

Entry	Polymer	Di-acid (mol%)						
		C4	C5	C6	C7	C8	C9	C10
1.	PE-4,4	100	-	-	-	-	-	-
2.	PE-4,5	-	100	-	-	-	-	-
3.	PE-4,6	-	-	100	-	-	-	-
4.	PE-4,7	-	-	-	100		-	-
5.	PE-4,8	-	-	-	-	100		-
6.	PE-4,9	-	-	-	-	-	100	-
7.	PE-4,10	-	-	-	-	-	-	100
8.	PE-4,5±1	25	50	25	-	-	-	-
9.	PE-4,6±1	-	25	50	25	-	-	-
10.	PE-4,7±1	-	-	25	50	25	-	-
11.	PE-4,8±1	-	-	-	25	50	25	-
12.	PE-4,9±1	-	-	-	-	25	50	25
13.	PE-4,6±2	11.11	22.22	33.33	22.22	11.11	-	-
14.	PE-4,7±2	-	11.11	22.22	33.33	22.22	11.11	-
15.	PE-4,8±2	-	-	11.11	22.22	33.33	22.22	11.11
16.	PE-4,7±3	6.25	12.5	18.75	25	18.75	12.5	6.25
17.*	PE-BHET,6±2	11.11	22.22	33.33	22.22	11.11	-	-
18.*	PE-BHET,8±2	-	-	11.11	22.22	33.33	22.22	11.11

*The diol was replaced by bis-hydroxyethyl terephthalate (BHET)

3. Biodegradability setup and calculations

Biodegradability experiments were performed on PE-4,6±Y, with Y = 0, 1 and 2. The bio-degradability experiments were performed in line with ASTM method D5988 – 18 for determining aerobic biodegradation of plastic materials in soil. A schematic overview of our experimental setup is shown in Figure S1. The amount of mineralization was determined by the amount of CO_2 uptake in a 0.5 M KOH solution (equation 1), which is titrated with a 0.25 M HCl (equation 2) approximately every week. The formulas that were used to determine the amount of CO_2 evolution are mentioned below.

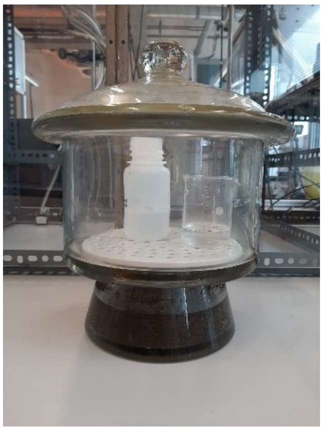

Figure S1. The experimental setup used for the biodegradability experiments. On the bottom, 500 g soil containing the plastic material with on top a beaker with 0.5 M KOH and a beaker with H_2O.

$$2\,KOH + CO_2 \rightarrow K_2CO_3 + H_2O \tag{1}$$

$$K_2CO_3 + 2\,HCl \rightarrow 2\,KCl + H_2CO_3 \tag{2}$$

Using the reaction equations 1 and 2, the concentration of an unknown KOH solution can be determined with equation 3. Then, based on initial and final KOH concentration, the amount of adsorbed CO_2 can be calculated with equation 4 and combining equation 3 and 5 gives equation 5.

$$Volume_{HCl} * [HCl] = Volume_{KOH} * [KOH] \tag{3}$$

$$[CO_2] = \frac{[KOH]_{initial} - [KOH]_{final}}{2} \tag{4}$$

$$[CO_2] = \frac{[KOH]initital - \frac{Volume_{HCl} * HCl}{Volume_{KOH}}}{2} \tag{5}$$

Subsequently, from the amount of adsorbed CO_2 and the known amount of carbon in the polymers it can be calculated what percentage of the polyester is mineralized (equation 6).

$$Weight\ loss\ (\%) = \frac{[CO_2]_{titrated}}{[C]_{polyester}} * 100\% \tag{6}$$

4. Characterization of the materials used for biodegradation

The biodegradability experiments were performed on a different batch of polyesters especially prepared for these experiments. Before biodegradation, the materials were characterized with thermogravimetric analysis (TGA) and differential scanning colorimetry (DSC) to confirm that the synthesis procedure was executed correctly. Subsequently, the number average molecular weight (M_n) was determined with nuclear magnetic resonance (NMR) by doing an end group analysis using equation 7. The M_n was determined using the -CO-O-**CH**$_2$- ester protons, which always gave a distinct signal, also for the mixed samples. As a result of the synthesis method, the end group is always alcohol and the -**CH**$_2$-OH protons were used for analysis. Subsequently, the number of repeating units can be obtained by dividing the intensity of four ester protons by the four end group protons. Then, the M_n could be determined from by multiplying the number of repeating units times the molar mass plus the mass of both end groups. The graphs (Figures S2 and S3) below show the NMR spectra of these polymers which were used to determine the M_n.

$$M_n = \frac{Intensity\ of\ the\ repeating\ unit}{Intensity\ end\ group} * 200.23\frac{g}{mol} + 2 * 73.11\frac{g}{mol} \qquad (7)$$

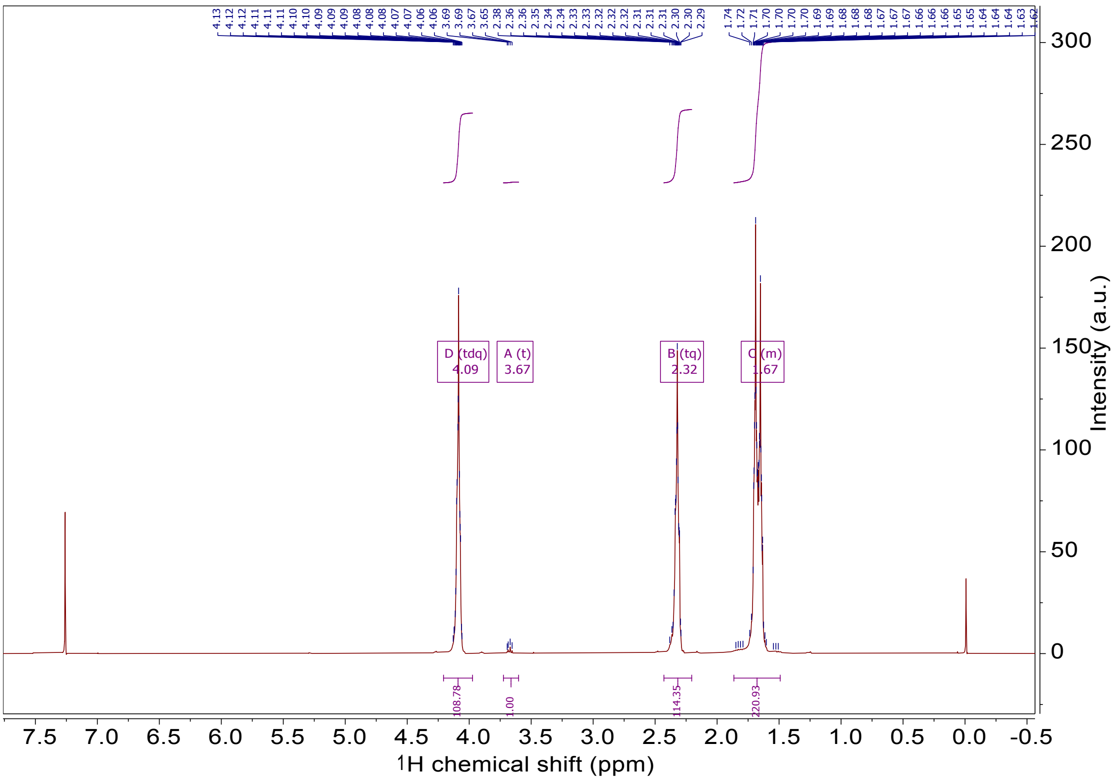

Figure S2. Nuclear magnetic resonance (NMR) spectrum of PE-4,6, which was used for the biodegradability experiments. End group analysis revealed a number average molar mass (M_n) of 21927 g/mol.

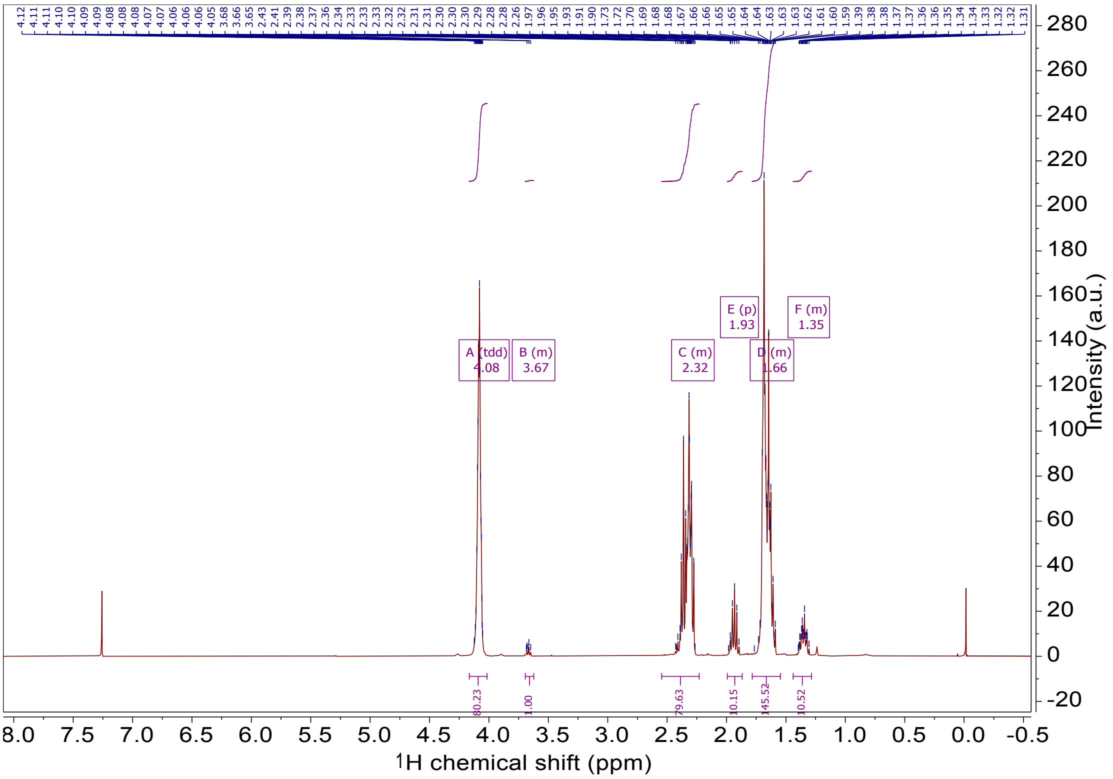

Figure S3. Nuclear magnetic resonance (NMR) spectrum of PE-4,6±1, which was used for the biodegradability experiments. End group analysis revealed a number average molar mass (M_n) of 16211 g/mol.

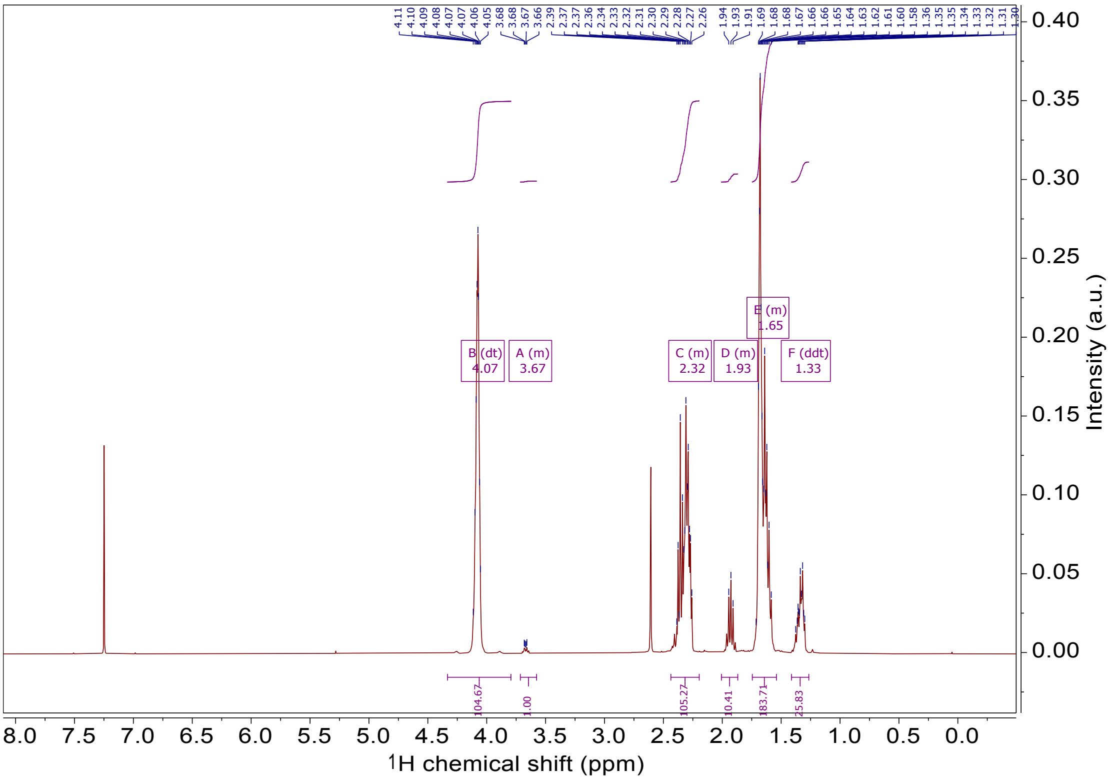

Figure S4. Nuclear magnetic resonance (NMR) spectrum of PE-4,6±2, which was used for the biodegradability experiments. End group analysis revealed a number average molar mass (M_n) of 21104 g/mol.

5. Gel permeation chromatography data

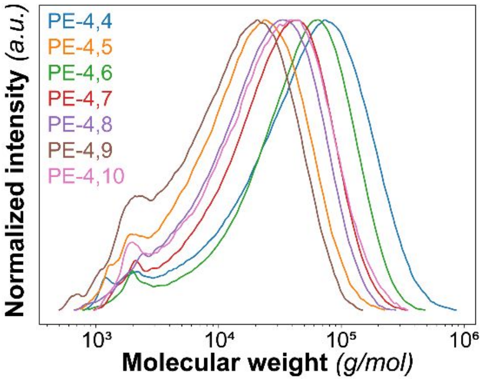

Figure S5. Gel permeation chromatography (GPC) profiles of the polyesters as function of di-carboxylic acid chain length.

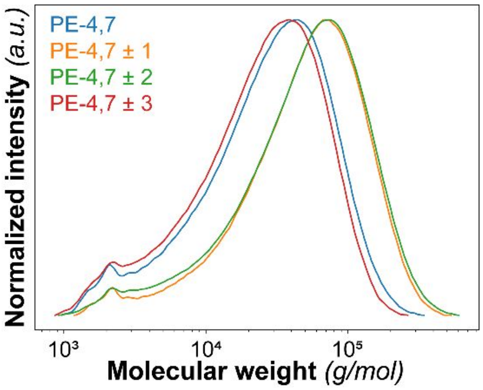

Figure S6. Gel permeation chromatography (GPC) profiles of PE-4,7 as function of mixing degree.

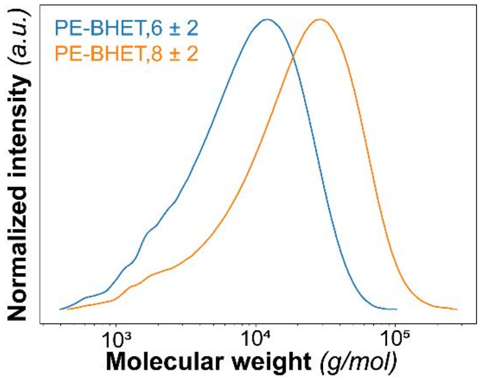

Figure S7. Gel permeation chromatography profiles of polyesters synthesized with BHET as the diol.

6. Differential scanning calorimetry and thermogravimetric analysis plots PE-4,6±Y and PE-4,8±Y

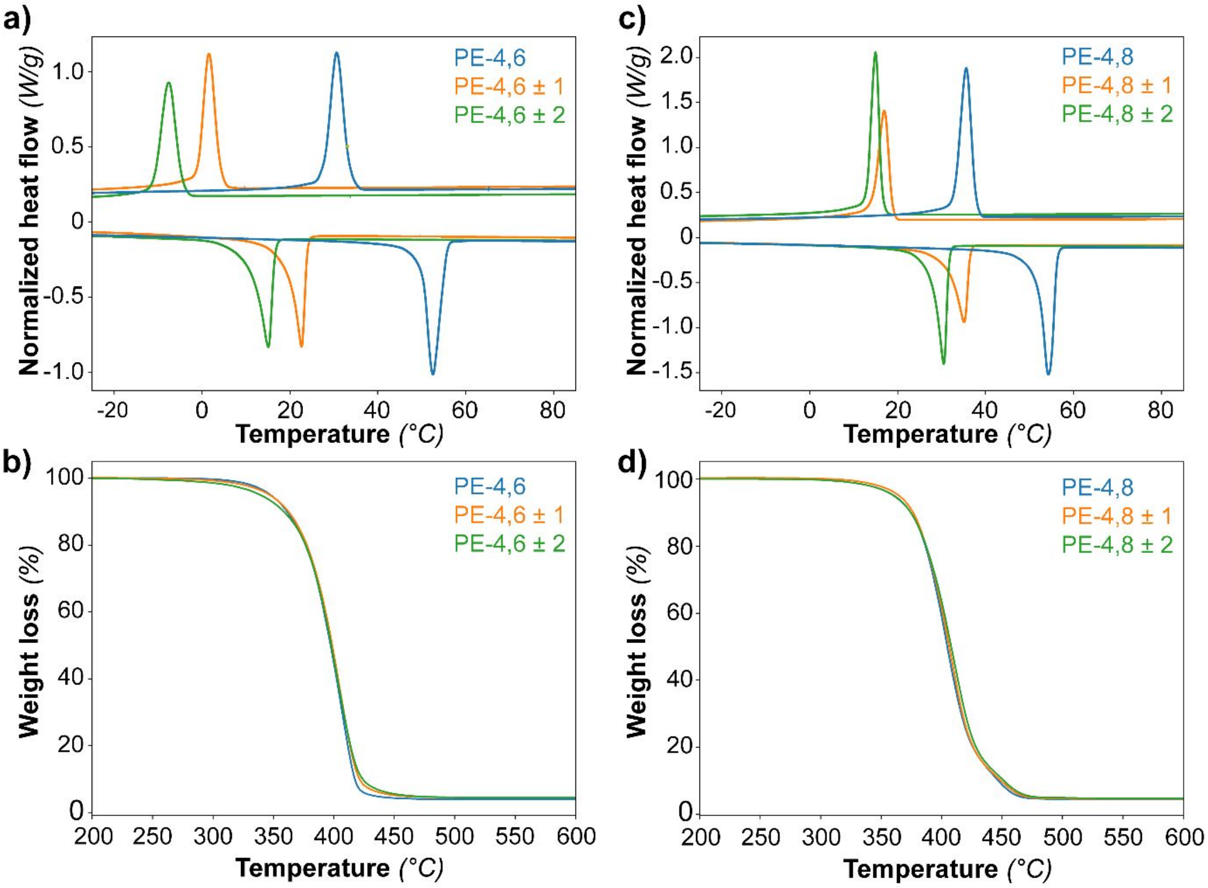

Figure S8 (a) Differential scanning calorimetry (DSC) curves of PE-4,6±y (exo up). (b) DSC curves of PE-4,8±y (exo up). (c) Thermogravimetric analysis (TGA) plots of PE-4,6±y. (d) TGA plots of PE-4,8±y.

7. Infrared spectra of the polyesters as function of chain length and mixing degree

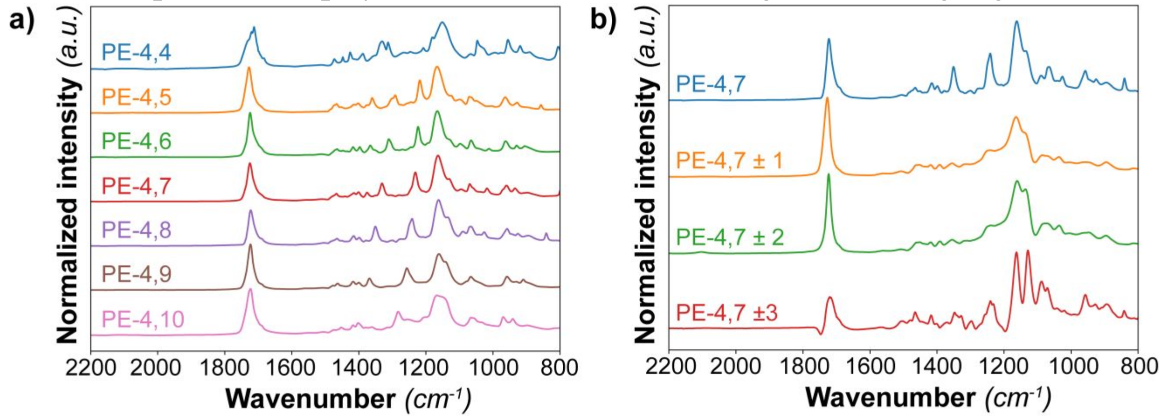

Figure S9. (a) Infrared (IR) spectra of the polyesters with composition PE-4,X as function of the di-acid chain length. (b) IR spectra of polyesters with composition PE-4,7±Y as function of mixing degree.

8. Infrared spectroscopy and differential scanning calorimetry data of the polyester derived from polyethylene

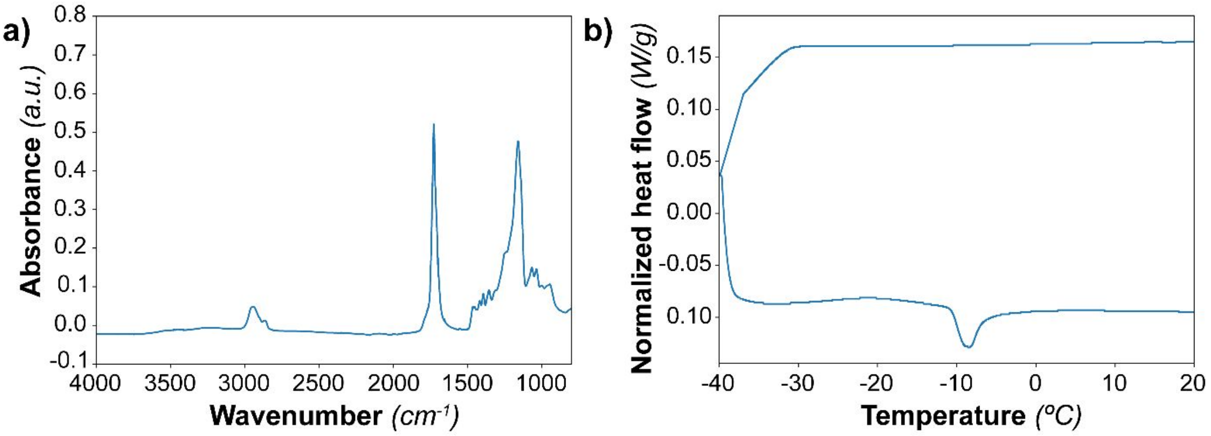

Figure S10. (a) Infrared (IR) spectrum and (b) differential scanning calorimetry data of the polyester prepared from di-carboxylic acids derived from high-density polyethylene (HDPE) and 1,4-butanediol.

9. Additional characterization polyesters prepared with BHET

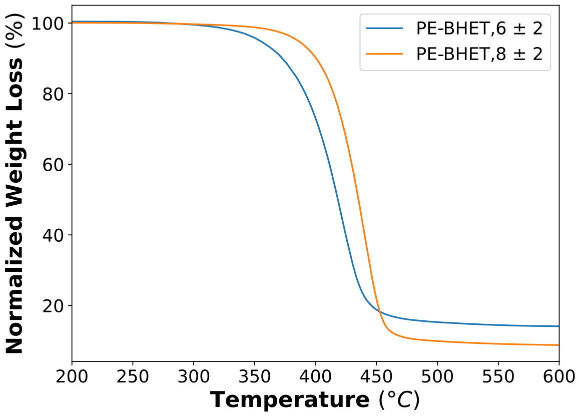

Figure S11. Thermogravimetric analysis (TGA) curves of polyesters produced with bis-hydroxyethyl terephthalate (BHET) and mixed di-carboxylic acids.

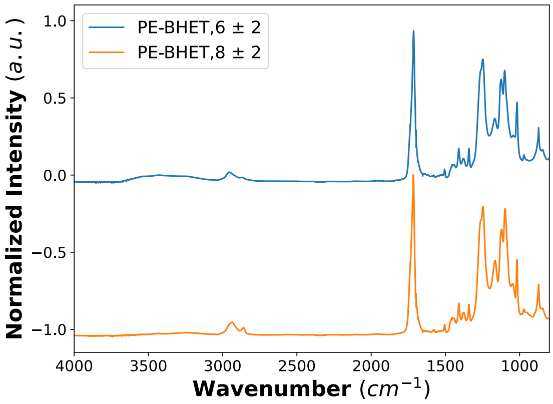

Figure S12. Infrared (IR) spectra of polyesters produced with bis-hydroxyethyl terephthalate (BHET) and mixed di-carboxylic acids.

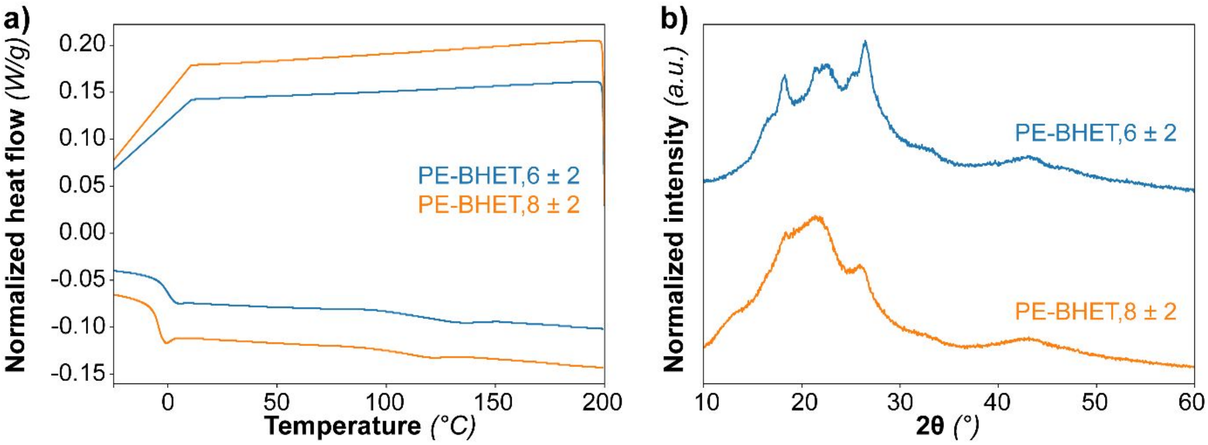

Figure S13. Characterization of two polymers prepared with bis-hydroxyethyl terephthalate (BHET) from a different batch. (a) Differential scanning calorimetry (DSC) curves of the polyesters produced with BHET (exo up). (c) X-ray diffraction (XRD) patterns of polyesters produced with BHET.

10. Detailed NMR assignment of the polyester derived from polyethylene

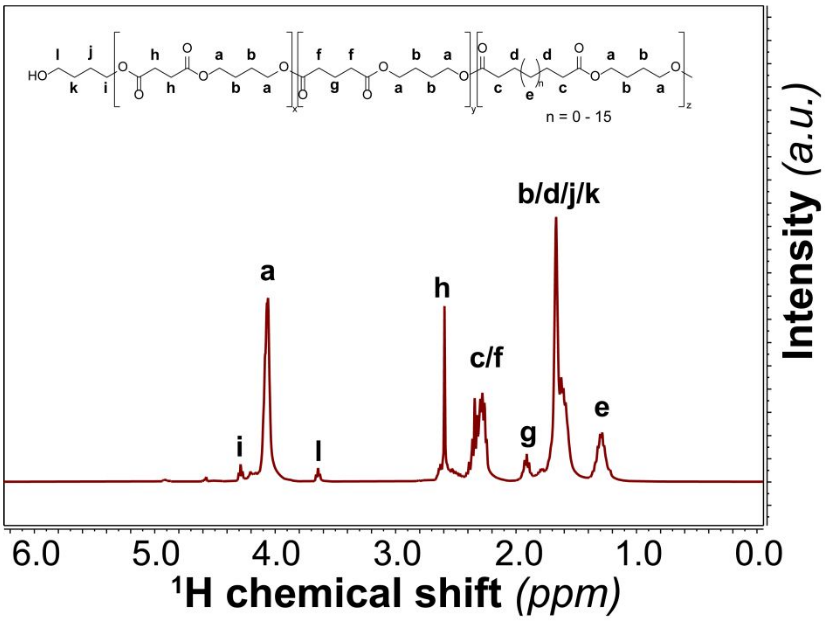

Figure S14. ^{1}H nuclear magnetic resonance (NMR) spectrum of the polyester synthesized from the di-carboxylic acid mixture derived from polyethylene with complete peak assignments.

11. Polyethylene oxidation: tuning di-carboxylic acid chain length and improving yield

The two-step polyethylene (PE) oxidation method described in the manuscript yielded di-carboxylic acids with a sufficient purity to obtain a polymer. However, the carbon recovery is low and HNO_3 is required. In addition, tunability of the di-carboxylic acid chain length distribution, depending on the application, would be desired. In Figure S15, we present two different methods to tune the average di-carboxylic acid chain length and improve their overall yield.

When PE was oxidized with O_2 in the presence 0.5 mol% with respect to -CH_2- of Mn stearate, the di-carboxylic acid yield can be improved to ~15 mol% (Figure S15a). Compared to the oxidation without a transition metal a significant reduction in the number of other chain end groups, such as γ-lactone, γ-ketone and methyl ketone was observed.[1] Furthermore, we observed a maximum in chain length distribution at around C10. The shift from most di-carboxylic acids being in the C4-6 range with aerobic or HNO_3 and Co/Mn/Br aided oxidation[2,3] to longer di-acids in the C10 range is interesting for polymer applications. Inspired by the work of Pifer and Sen, we conducted a PE oxidation experiment with O_2/NO (Figure S15b).[4] The additional NO leads to the formation of short-chain di-carboxylic acids (C4 – C8) in a yield of ~28 mol%. In addition, all typical side products observed for aerobic oxidation, e.g. methyl ketone, γ-lactone, γ-ketone,[1] were not observed. The main side-products were di-carboxylic acids (C4 – C8) bearing an additional nitro group. Both methods in combination with the existing literature in the field of PE oxidation show that the di-carboxylic acid chain length is somewhat tunable and that the yields can be improved. Nevertheless, these product mixtures still contain impurities and partially converted PE and suitable purification will need to be developed before the products can be polymerized.

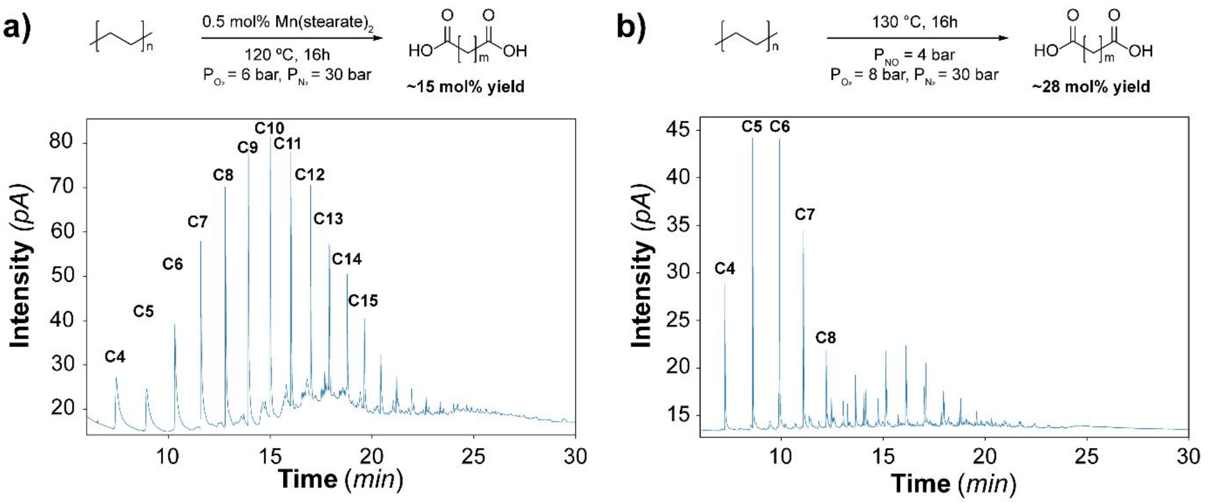

Figure S15. (a) Gas chromatography-flame ionization detection (GC-FID) chromatogram for PE oxidized with 0.5 mol% Mn(stearate)$_2$ at 120 ˚C for 16 h at a pressure of 30 bar synthetic air (P_{O2} = 6 bar, P_{N2} = 24 bar). (b) GC-FID chromatogram of PE oxidized at 130 ˚C for 16 h with O_2/NO_x (P_{O2} = 8 bar, P_{NO} = 4 bar, P_{N2} = 28 bar).

References

(1) Smak, T. J.; de Peinder, P.; Van der Waal, J. C.; Altink, R.; Vollmer, I.; Weckhuysen, B. M. Oxidative Conversion of Polyethylene Towards Di-Carboxylic Acids: A Multi-Analytical Approach. *ChemSusChem* **2024**, *17*, e202301198. https://doi.org/10.1002/cssc.202301198.

(2) Bäckström, E.; Odelius, K.; Hakkarainen, M. Trash to Treasure: Microwave-Assisted Conversion of Polyethylene to Functional Chemicals. *Ind. Eng. Chem. Res.* **2017**, *56*, 14814–14821. https://doi.org/10.1021/acs.iecr.7b04091.

(3) Partenheimer, W. Valuable Oxygenates by Aerobic Oxidation of Polymers Using Metal / Bromide Homogeneous Catalysts. *Catal. Today* **2003**, *81*, 117–135. https://doi.org/10.1016/S0920-5861(03)00124-X.

(4) Pifer, A.; Sen, A. Chemical Recycling of Plastics to Useful Organic Compounds by Oxidative Degradation. *Angew. Chem. Int. Ed.* **1998**, *37*, 3306–3308. https://doi.org/10.1002/(SICI)1521-3773(19981217)37:23<3306::AID-ANIE3306>3.0.CO;2-B.
